# Intratumour microbiota modulates adrenocortical cancer responsiveness to mitotane

**DOI:** 10.1530/ERC-23-0094

**Published:** 2023-09-11

**Authors:** Giulia Cantini, Elena Niccolai, Letizia Canu, Leandro Di Gloria, Simone Baldi, Arianna Pia Propato, Laura Fei, Giulia Nannini, Soraya Puglisi, Gabriella Nesi, Matteo Ramazzotti, Amedeo Amedei, Michaela Luconi

**Affiliations:** 1 Department of Experimental and Clinical Biomedical Sciences, Endocrinology Section, University of Florence, Florence, Italy; 2 European Network for the Study of Adrenal Tumors (ENS@T) Center of Excellence, Florence, Italy; 3 Centro di Ricerca e Innovazione sulle Patologie Surrenaliche, AOU Careggi, Florence, Italy; 4 Istituto Nazionale Biostrutture e Biosistemi (I.N.B.B.), via delle Medaglie D’Oro, Rome, Italy; 5 Department of Experimental and Clinical Medicine, University of Florence, Florence, Italy; 6 Department of Experimental and Clinical Biomedical Sciences, Biochemical Sciences Section, University of Florence, Florence, Italy; 7 Department of Clinical and Biological Sciences, Internal Medicine, San Luigi Gonzaga Hospital, University of Turin, Orbassano, Turin, Italy; 8 Department of Health Sciences, Pathology Section, University of Florence, Florence, Italy

**Keywords:** microbiota, oncobiome, tumour microenvironment, rare endocrine tumours, bacteria

## Abstract

The infiltrating microbiota represents a novel cellular component of the solid tumour microenvironment that can influence tumour progression and response to therapy. Adrenocortical carcinoma (ACC) is a rare and aggressive endocrine malignancy for which mitotane (MTT) treatment represents the first-line therapy, though its efficacy is limited to a therapeutic window level (14–20 mg/L). Novel markers able to predict those patients who would benefit from MTT therapy are urgently needed to improve patient’s management. The aim of our study was to evaluate the presence of intratumoural bacterial microbiota DNA in 26 human ACC tissues vs 9 healthy adrenals; moreover, the association between the relative bacterial composition profile, the tumour mass characteristics and MTT ability to reach high circulating levels in the early phase of treatment, were explored. We found the presence of bacterial DNA in all adrenal samples from both tumours and healthy cortex specimens, documenting significant differences in the microbial composition between malignancy and normal adrenals: in detail, the ACC tissues were characterised by a higher abundance of the Proteobacteria phylum (especially the *Pseudomonas* and *Serratia* genera). In addition, the Proteobacteria’s low abundance was negatively associated with tumour size, Ki67 and cortisol secretion. MTT levels reached higher levels at 9 months in ACC patients with high abundance of Proteobacteria, *Pseudomonas* and *Serratia* and with low abundance of Bacteroidota, Firmicutes and *Streptococcus*. These findings are the first indication that human ACCs are characterised by infiltrating bacteria and their specific abundance profile seems to influence the increase in circulating MTT levels at 9 months.

## Introduction

Recent studies allowed the identification and characterisation of cancer-type-specific profiles of bacteria associated with tumour and infiltrating immune cells, resulting in the current concept that microbiota represents a new cellular component of the tumour microenvironment (TME) (Nejman *et al.* 2020, Sepich-Poore *et al.* 2021, Ciernikova *et al.* 2022, Gao *et al.* 2022). In particular, it is clearly emerging that the intratumoural microbiota (ITM), the oncobiome (Thomas & Jobin 2015, Di Gloria & Niccolai 2022), actively interacts with the cancer cells and TME, modulating tumour response and progression (Geller *et al.* 2017) through regulating the local immune infiltrate and inducing innate and adaptive immune suppression (Pushalkar *et al.* 2020, Hezaveh *et al.* 2022). Notably, bacterial load as well as differences in the taxa profiles of the cancer-associated microbiota can be correlated with clinical characteristics, tumour behaviour (Gao *et al.* 2022) and response to therapies (Nejman *et al.* 2020, Qiao *et al.* 2022).

Adrenocortical carcinoma (ACC) is a rare endocrine malignancy characterised by high aggressiveness and poor prognosis, in particular when metastatic at diagnosis. Mitotane (MTT) oral treatment is recommended in patients with advanced disease, as monotherapy or in combination with etoposide, doxorubicin and cisplatin (EDP), and also alone in adjuvant settings after surgical resection (Fassnacht *et al.* 2018). However, its efficacy depends on reaching the blood concentration of the therapeutic window (14–20 mg/L, Haak *et al.* 1994). The time to reach this range does not seem to be influenced by the type of therapeutic regimen (Kerkhofs *et al.* 2013), but polymorphisms in *Cyp* genes are associated with a significantly higher increase in blood concentration in the early phases (Altieri *et al.* 2020). Notably, relevant limitations of MTT treatment are associated with the presence of non-responders and a high risk of treatment discontinuation for severe toxicity (Puglisi *et al.* 2020). Therefore, markers able to predict not only patients who would benefit from MTT but also those rapidly reaching the therapeutic doses are urgently needed to improve patient’s management.

Since the ITM has been described to have an impact on solid tumours’ response to therapies, we were interested in assessing the presence of intratumoural bacterial components in ACC patients as well as any possible association between specific ITM composition and MTT circulating levels.

This is the first study to report the presence of a resident bacterial microbiota in adrenals, showing a different composition in ACC compared to non-tumoural conditions.

## Materials and methods

### Patients and ethical approval

We retrospectively analysed a series of 26 conventional ACCs following the surgical removal of the tumour at Careggi University Hospital, Florence, Italy, between 2007 and 2021. Formalin-fixed paraffin-embedded tumour samples were available for immunohistochemical analysis. The study design was reviewed and approved by the Careggi University Hospital Ethical Committee (Prot. 2011/0020149 – Rif CEAVC Em. 2019-201 26/11/2019). The patients recruited gave their written informed consent. Healthy adrenals (*n* = 9) were obtained and collected from healthy donors during nephrectomy or following cadaveric explants (Prot. 2011/0020149 – Rif CEAVC Em. 2019-201 26/11/2019) and snap frozen and stored at −80°C for subsequent metagenomic analysis.

### Pathologic analysis of ACC samples

Histologic ACC diagnosis was carried out by two independent reference pathologists on tumour tissue removed at surgery. Tumour size was measured at surgery, and functional activity was measured routinely by mass spectrometry at Careggi University Hospital on blood samples obtained before surgery. Tumour specimens were evaluated according to the Weiss scoring system, in which the presence of three or more criteria highly correlates with malignant behaviour (WHO 2022). The Ki67 labelling index (LI) was estimated on digitised glass slides, after immunohistochemical staining with anti-human Ki67 antibody (1:40 dilution, MIB-1, Dako) (Poli *et al.* 2019). Areas with the highest labelling were manually identified, Ki67-positive nuclei were counted in 1000 tumour cells and Ki67 LI was expressed as the percentage of labelled cells, using the Ki67 algorithm available in the Picture Archiving and Communication System (PACS) (Sectra Medical, Linkoping, Sweden). Tumour stage was assessed according to the revised 8th edition of the TNM classification of ACC proposed by the European Network for the Study of Adrenal Tumours (ENSAT) (Fassnacht *et al.* 2009).

### Mitotane treatment and drug level measurements

For MTT treatment, all patients received the same MTT formulation, Lysodren^®^ 500 mg tablets for ≥6 months and measurements were performed between 3 months from the start of the treatment and the end of therapy ranging from 24 to 60 months. The measurements obtained between 3 and 24 months were included in the study. The low-dose approach was prescribed with a starting dose of 1 g/day increased every 3 days by 0.5 g up to 3 g/day and then adjusted according to MTT levels and tolerability (Terzolo *et al.* 2000). MTT concentrations were retrieved from the Lysosafe Online database (www.lysosafe.com). Lysosafe Online^®^ is a login-protected website that stores MTT plasma concentrations of patients treated by physicians who have registered with the Lysosafe^®^ service, a free-of-charge service of measurement of plasma MTT concentrations in ACC patients offered by HRA Pharma to European prescribers since 2005 and associated with the use of Lysodren^®^. Samples were sent to a centralised laboratory, extracted by precipitation with ethanol and tested by a standardised gas chromatography/mass spectrometry method. Plasma MTT values of any patient are available for the treating physician on Lysosafe Online, in a historical and graphic plot that matches MTT levels with the relative Lysodren^®^ dose. Patient data are anonymous during the whole process since patients are recorded using an acronym consisting of initials combined with date of birth.

### Metagenomic analysis

Genomic DNA was extracted from the 26 ACC and 9 healthy adrenal frozen biopsies using DNeasy Blood & Tissue Kit (Qiagen). The quality and quantity of extracted DNA was assessed using the NanoDrop ND-1000 (Thermo Fisher Scientific) and the Qubit 2.0 Fluorometer (Thermo Fisher Scientific, Waltham, MA, USA), respectively.

### 16S sequencing and bioinformatics analysis

DNA extracted from the 26 ACC and 9 healthy adrenal samples were sent to IGA Technology Services (Udine, Italy) where amplicons of the variable V3–V4 region of the bacterial 16s rRNA gene were sequenced using a paired-end approach (2 × 300 cycles, 50,000 reads) on the Illumina MiSeq platform according to the Illumina 16S Metagenomic Sequencing Library Preparation protocol.

Sequencing results were analysed using QIIME2 2022.8. The sequencing primers and the reads without primers were removed using the Cutadapt tool. DADA2 was used to perform paired-end reads filtering, merging and chimeras removal steps after trimming low-quality nucleotides from both forward and reverse reads while ensuring a 20 nt long overlap region. Hence, ASVs (amplicon sequence variants) were generated and those potentially derived from the host DNA were identified through alignment to the GRCh38 database with Bowtie2 2.2.5 and then discarded. Taxonomic assignments were performed through the Scikit-learn multinomial naive Bayes classifier re-trained on SILVA database (release 138) V3-V4 iper-variable region. In conclusion, every genus with a mean relative abundance less than 0.05% (computed considering also the host DNA in the total library size) has been removed to further minimise environmental contaminants. Further details are openly available in the related R script (see Data availability section).

### Statistical analysis

Continuous variables with normal distribution were presented as mean (standard deviation (SD)) and nonparametric variables as median (interquartile range (IQR)). Categorical variables were expressed as counts and percentages. Statistical analysis was performed with SPSS 28.0 (Statistical Package for the Social Sciences) for Windows. A *P*-value less than 0.05 was considered statistically significant. Possible associations were investigated using the *χ* ^2^ test for categorical variables and Spearman’s correlation for continuous variables. Comparison between two groups of data was accomplished using Student’s *t-*test for normally distributed variables and Mann–Whitney *U* test for nonparametric variables or paired Wilcoxon’s signed-rank test.

Regarding the analyses on bacterial communities, the statistical analyses were performed in R 4.2 with the help of packages phyloseq 1.38.0, DESeq2 1.36.0 and other packages satisfying their dependencies as vegan 2.6-2. Packages ggplot2 3.3.5, ggh4x 0.2.2 and dendextend 1.15.2 were used to plot data and results. A saturation analysis on raw ASV was performed on every sample using the function rarecurve (step 100 reads), further processed to highlight saturated samples (arbitrarily defined as saturated samples with a final slope in the rarefaction curve with an increment in ASV number per reads <1e−5) in order to check the sample ASV saturation.

Shannon index, Observed ASV richness and Pielou’s evenness were used to estimate bacterial diversity in each sample using the function estimate richness from phyloseq. The Pielou’s evenness index was calculated using the formula E = S/log(R), where S is the Shannon diversity index and R is the number of ASVs in the sample. Differences in all indices were tested using the Wilcoxon’s test. Principal coordinates analyses (PCoAs) were performed using the Hellinger distance on Hellinger transformed genera abundances to address the compositionality of those data (Legendre & Legendre 2012). At the different taxonomic ranks, the differential abundances (DA) have been computed through the DESeq2 algorithm on raw count data. The DA with an associated *P*-value (adjusted through Benjamini–Hochberg method) less than 0.05 has been considered significant. Moreover, every DA with a grand mean count <50 has been discarded from the displayed results to avoid the most likely noisy ones. Further details are openly available in the related R script (see Data availability section).

## Results

We performed 16S rRNA gene sequencing in DNA extracted from fresh frozen biopsies from *n* = 26 patients affected by ACC and adrenal tissues from 9 healthy controls (HC). Patients’ and tumours’ characteristics are reported in Table 1. Bacterial DNA was found in all the tissue specimens analysed. We sequenced a total of 1,738,846 reads from 35 adrenal samples. After the removal of human amplicons and other bioinformatics pre-processing steps, 717,724 (41.3%) reads were available for further analysis.

**Table 1:** Tumour characteristics in the ACC patients’ cohort. Data are expressed as mean ± SD for continuous parametric and median (interquartile) for non-parametric variables and as absolute number and percentage of patients for non-continuous variables.

ACC patient cohort (*n*= 26)	
Secretion^a^ (%)	
Cortisol	13 (50)
Androgens	5 (19)
Aldosterone	1 (4)
Non-secreting	8 (31)
NA	1 (4)
Tumour diameter (cm)	9.7 ± 5.0
*ENS@T Stage (%)*	
I	7 (27)
II	6 (23)
III	9 (35)
IV	4 (15)
Total Weiss score	6 (4–8)
Ki67 LI	15.0 (5.0–22.5)
RMS (%)	
R0	21 (81)
R2	5 (19)

^a^Multiple secretions are indicated as independent. Ll, labelling index; NA, not available; RMS, resection margin status (R0 – complete resection, R2 macroscopic residual disease).

**Figure 1:**
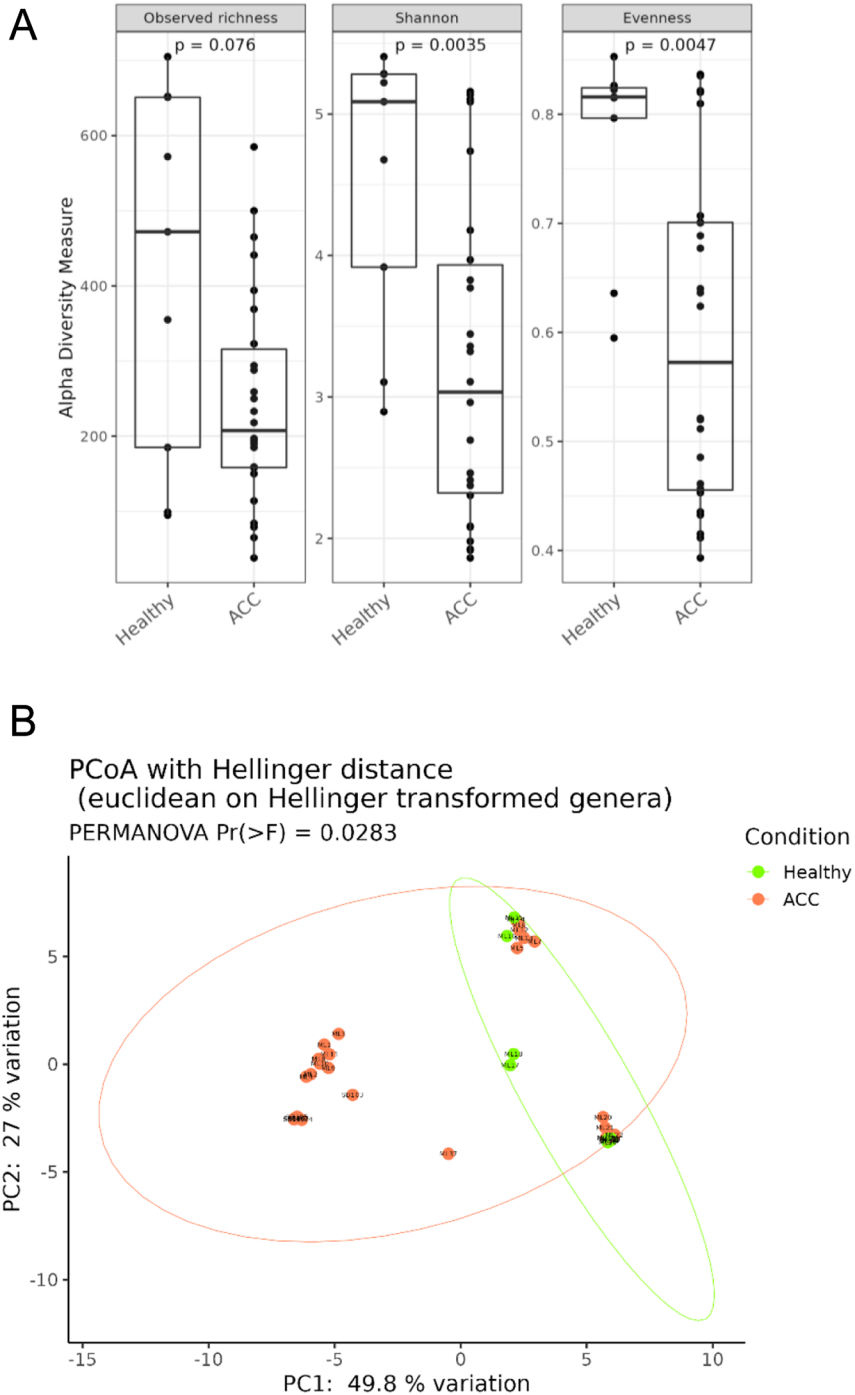
(A) Boxplots showcasing alpha diversity indices (Observed richness, Shannon index and Evenness) in ACC and healthy adrenal tissues (healthy). Statistical differences were evaluated using paired Wilcoxon’s signed-rank test. *P*-values less than 0.05 were considered statistically significant. (B) Principal coordinates analysis (PCoA) according to the Bray–Curtis beta diversity metric. Results of the permutational multivariate analysis of variance (PERMANOVA) are also shown based on the first two coordinates. A full colour version of this figure is available at https://doi.org/10.1530/ERC-23-0094.

The analysis of alpha diversity, estimated by Observed richness, Shannon and the evenness indexes, reveals that the microbial diversity was significantly low and less homogeneously distributed in ACC compared to HC (Shannon: *P* = 0.0035; Evenness: *P* = 0.0047). Furthermore, as regards the beta diversity calculated through PCoA using Hellinger distance on genera, a significantly different microbial composition was documented (PERMANOVA = 0.0283) (Fig. 1).

**Figure 2:**
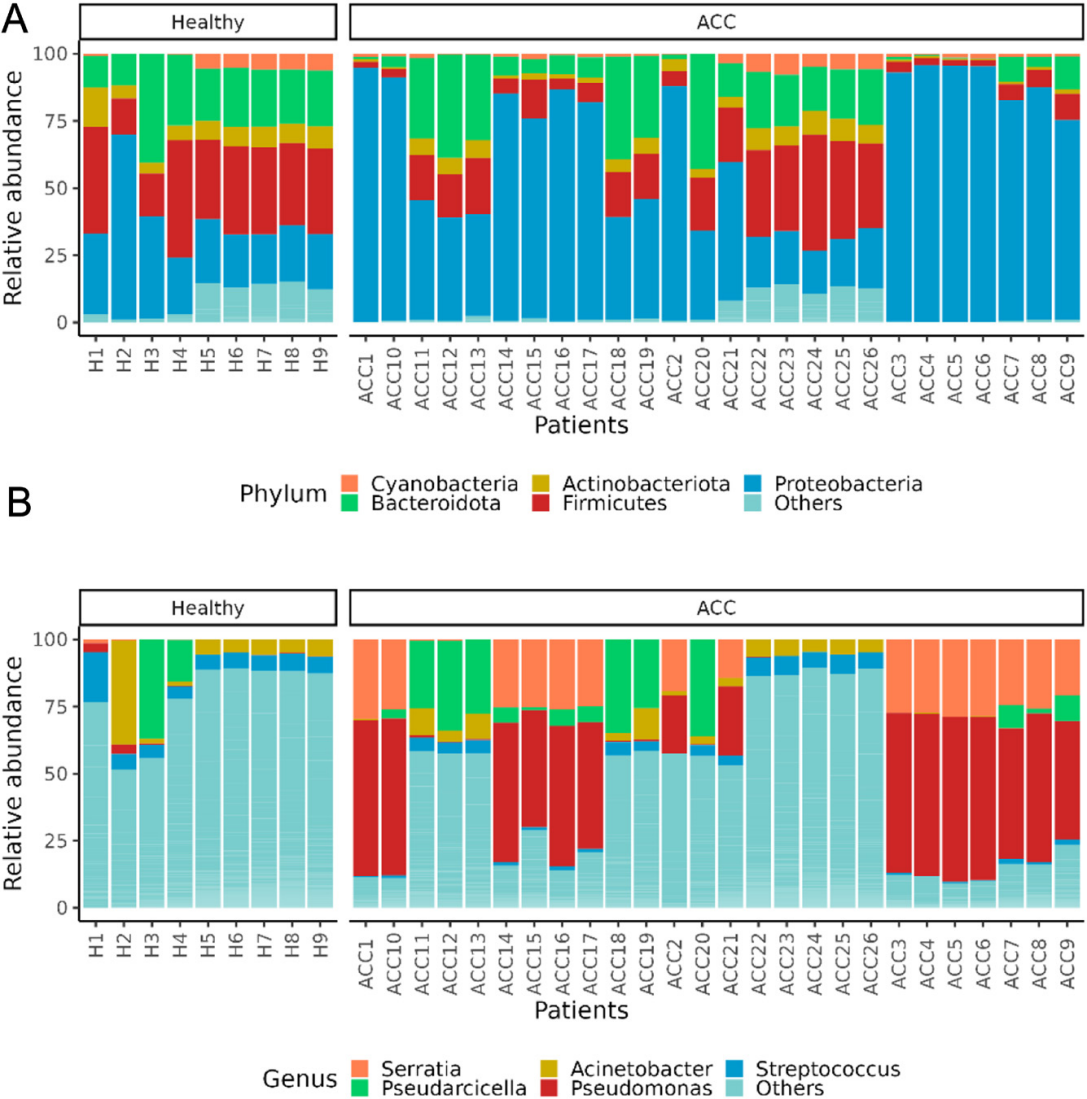
Bar plot showing the relative abundances (%) of the top 5 phyla (A) and genera (B) in ACC and healthy adrenals (Healthy). Twenty-six ACC samples from 26 different patients were analysed as well as healthy adrenal tissue from nine different subjects (H); ‘others’ groups include every phylum (A) or genus (B) below rank 5.

The taxonomic composition analysis revealed that five phyla were predominant in all samples (ACC and HC): Proteobacteria (53.36%), Firmicutes (18.52%), Bacteroidota (16.49%), Actinobacteriota (4.62%) and Cyanobacteria (2.38%), while the five most abundant genera were *Pseudomonas* (23.10%), *Serratia* (17.11%), *Pseudarcicella* (17.28%), *Acinetobacter* (4.24%) and *Streptococcus* (4.02%) (Fig. 2).

**Figure 3:**
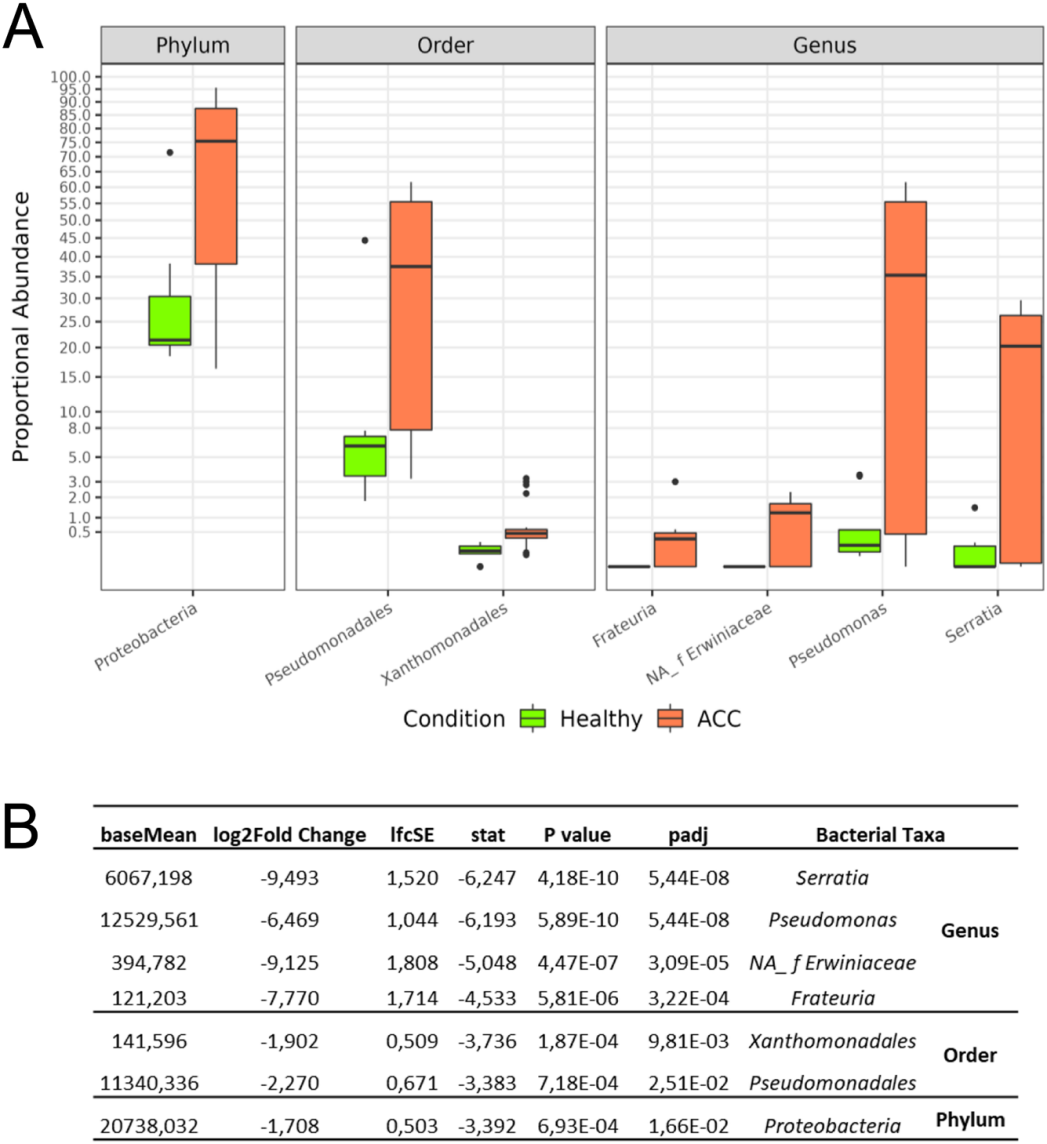
Statistically significant differences among ACC and healthy adrenals. (A) Boxplot showing the abundance of phyla, orders and genera associated with a statistically significant variation. (B) DESeq2 results of the differential abundance taxa in ACC patients compared to healthy adrenals (healthy); adjusted *P* values (adjusted through Benjamini–Hochberg method) are indicated under the column ‘padj’; baseMean, the average count values normalised by size factors; lfcSE, log2FoldChange standard error; stat, log2FoldChange/lfcSE (the Wald statistic); *NA_f* Erwiniaceae, non-assigned genus ofErwiniaceaefamily. A full colour version of this figure is available at https://doi.org/10.1530/ERC-23-0094.

Univariate analyses, performed to identify bacterial taxa that differed significantly between the two groups (ACC vs HC samples), revealed a significant increase in the abundance of the following bacteria in the ACC group compared to the HC group: at the phylum level, Proteobacteria (log2FC = 1.70, *P*-adj = 0.02); at the order level, Pseudomonadales (log2FC=2.27, *P*-adj = 0.02) and Xanthomonadales (log2FC = 1.90, *P*-adj = 0.01); at the genus level, *Frateuria* (log2FC = 7.77, *P*-adj < 0.0001), *Pseudomonas* (log2FC = 6.47, *P*-adj < 0.0001), *Serratia* (log2FC = 9.49, *P*-adj = <0.0001) and an unclassified genus of Erwiniaceaefamily (log2FC = 9.12, *P*-adj < 0.0001) (Figs. 2B, 3A and B).

Once identified the abundance profiles of intratissue bacteria that differ between ACC and healthy adrenocortex conditions, we focused on ACC samples and investigated if there were differences among ACC patients based on the clinico-pathologic features.

The relevant clinico-pathologic characteristics of ACC patients (*n* = 26) are illustrated in Table 1. Our cohort included 50% males, with a mean ± SD age of 50 ± 12 years. Median (IQR) follow-up was 57.5 (36.2–73.5) months and the time to progression (TtP) was 45.5 (24.5–72.7) months. Overall, 19% of the patients died from the disease, 39% experienced recurrence and 27% experienced progression.

The distributions of microbiota relative composition at the level of the most represented phyla and genera found in ACC tissue are reported in Table 2.

**Table 2:** Composition of intratumoural microbiota in ACC. Data represent the percentage of the relative ITM abundance and are expressed as the median percentage (33rd–66th percentile range) in top 5 phyla and genera found in the 26 ACC biopsies analysed.

Top five phyla		Top five genera	
Proteobacteria	74.4 (38.5–86.3)	Pseudomonas	34.7 (0.6–52.0)
Bacteroidota	10.7 (5.2–19.2)	Serratia	20.1 (0.1–25.4)
Firmicutes	12.1 (5.8–16.8)	Pseudarcicella	1.4 (0.0–6.2)
Actinobacteriota	2.8 (1.1–6.0)	Acinetobacter	1.0 (0.1–4.2)
Cyanobacteria	1.2 (1.0–1.5)	Streptococcus	1.9 (1.2–4.3)

The analyses of microbial richness and taxa distribution did not indicate significant differences between patients stratified according to secretion (yes or not), tumour size (cut-off = up to 6 cm or more) and Weiss score (cut off = 6 up to 6 or higher) (data are not shown).

We then stratified continuous parameters by dichotomisation in low and high abundance of each distribution on the basis of the 33rd percentile taken as the cut-off value.

When exploring the occurrence of association between microbiota composition and the clinical characteristics of ACC tumours, only Proteobacteria phylum and *Serratia* genus displayed a significant association with tumour size (*r* = −0.389, *P* < 0.05 and *r* = −0.572, *P* < 0.005, respectively) and negative relation with Weiss score class (cut-off < 6, *χ*^2^ = 4.4, *P* < 0.05 and *χ*^2^ = 5.5, *P* < 0.01, respectively); cortisol secretion was associated with Proteobacteria low composition (*χ*^2^ = 4.4, *P* < 0.05). No additional association was found with Ki-67 or tumour stage (not shown).

Focusing on the parameters associated with the MTT response in patients under MTT therapy (*n* = 17/26, 65%), we found a significant correlation between MTT early plasma levels, in particular at month 9 from the treatment’s start, and specific microbiota distribution in the ACC specimens.

**Figure 4:**
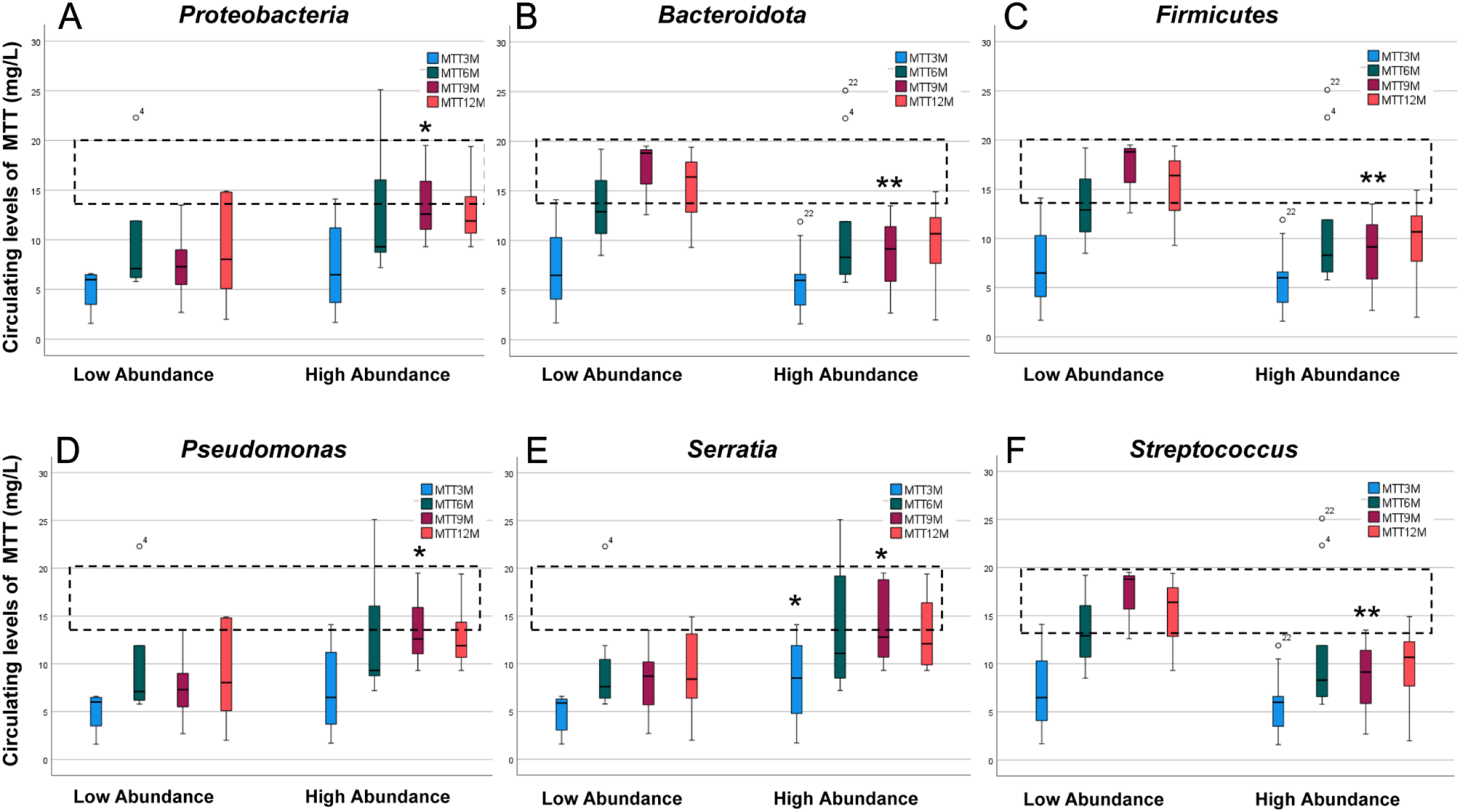
Boxplot showing the distribution of circulating levels of MTT at different time intervals of treatment according to intratumoural taxa composition. MTT levels have been measured by Lysosafe procedure in blood samples drawn at the indicated time intervals from the beginning of the treatment. Stratification of different microbiota taxa in low and high abundance classes was performed using the 33rd percentile of taxa distribution as cut-off (38.4%, 5.2%, 5.8% for Proteobacteria, Bacteroidota, Firmicutes, respectively; 0.6%, 1.0%, 1.2% for *Pseudomonas, Serratia* and *Streptococcus*, respectively). Dotted belts indicate the therapeutic window for MTT (14–20 mg/L). Student’s *t*-test for parametric distributions, with *P* values: ^*^P < 0.05 and^ **^P < 0.01 low vs high abundance.

Stratification of patients according to low and high composition of intratumoural bacteria (using the 33rd percentile of the distribution as cut-off, see Table 2), we found that MTT reached significantly higher levels at 9 months in patients with tumours characterised by high abundance of Proteobacteria and conversely with low abundance of Bacteroidota and Firmicutes at the level of phyla (Fig. 4A, B and C); whereas considering genera distribution, higher MTT levels were found in those patients where tumours showed a high composition of *Pseudomonas* and *Serratia* or where *Streptococcus* abundance was low (Fig. 4D, E and F).

## Discussion

To the best of our knowledge, this represents the first report documenting the resident bacteria oncobiome in the adrenal cortex, with specific profiles associated with malignancy. A peculiar bacterial composition is associated with MTT reaching high circulating levels in the first 9 months of treatment, as well as with some tumour characteristics.

Our report contributes to the finding of bacterial DNA in solid tumours that develop in organs with no direct connection with the external environment as the adrenals, in addition to what has already been described for ovarian cancer, glioblastoma multiforme and bone cancer (Neijman *et al.* 2020).

Although intratumoural microbiota, composed not only of bacteria but also of fungi and viruses, has largely been described as associated with a variety of solid tumours (Neijman *et al.* 2020, Sepich-Poore *et al.* 2021), the microbial vs tumour cell mechanisms and microbiota interactions in carcinogenesis still remain debated. In fact, it is still an open question if the oncobiome is only a bystander or if it can actively modulate the tumour progression and response to drugs, as well as the immunocompetence in cancer. In addition, potentially, the bacterial antigens specifically expressed by infected tumour cells may represent a selective target for novel anti-tumour therapies involving vaccines and CAR-T-cell strategies.

The presence of resident microbiota in several tissues and tumours not necessarily exposed to the external environment may suggest colonising abilities of microbiota and an active role of some species functional to the organism when in physiological conditions. This equilibrium is altered by the tumour that seems to modify the local microbiota profile in a reciprocal crosstalk that contributes to cancer progression. Interestingly, the specific microbiota profiles seem not only related to the histological characteristics of the organs where the tumour originates, but they also display a heterogeneous distribution inside the tumour mass (Wang *et al.* 2021), expanding the concept of tumour sub-clonality to the oncobiome where micro-niches of bacteria would influence cancer sub-clonality (Galeano Niño *et al.* 2022, Sepich-Poore *et al.* 2022).

The technique used to detect bacterial DNA did not enable us to quantify the number of bacteria present in the tissue or specify their intra- or extracellular localisation but to assess only the relative composition of taxa in the tissue.

Among the phyla highly represented in adrenals, we found significant differences between normal adrenals and ACC for Proteobacteria, confirming similar findings previously obtained in tissue of breast cancer (Thompson *et al.* 2017).

The high abundance of Proteobacteria is associated with better prognostic characteristics of ACC, such as reduced size, no excess of cortisol secretion and decreased Weiss Score; thus, Proteobacteria composition may represent a protective factor against tumour development.

In addition, the association between MTT levels in ACC patients and intratumoural bacteria showed that higher levels of MTT were reached between 6 and 9 months of treatment in the presence of a high abundance of Proteobacteria, in particular, *Pseudomonas* or *Serratia* genera, while a low presence of Firmicutes, in particular the genus *Streptococcus*, was associated with high drug levels.

Notably, the therapeutic range of MTT (14–20 mg/L) was not reached in the 3- to 12-month interval from the beginning of treatment either when Proteobacteria (Fig. 4A), *Pseudomonas* (Fig. 4D) and *Serratia* (Fig. 4E) were low represented or when Bacteroidota (Fig. 4B), Firmicutes (Fig. 4C) or *Streptococcus* (Fig. 4F) were highly abundant. These data suggest the existence of specific abundance profiles for some microbiota which are permissive or non-permissive for MTT. Notably, some infiltrating bacteria have been associated with protective effects vs solid tumours (Chen *et al.* 2022) through the secretion of specific cytotoxic factors, including the genus *Serratia* (Araghi *et al.* 2019, Guryanov *et al.* 2020) in CRC and *Pseudomonas* in hepatocarcinoma (Qu *et al.* 2022). Intratumoural colonisation by specific bacterial strains has previously been suggested to modulate the levels and activity of chemotherapy by active metabolisation of the drugs (LaCourse *et al.* 2022), as described for colorectal carcinoma, where some bacteria have the ability to internalise and detoxify 5-FU, likely through dedicated nucleoside import and pyrimidine scavenging pathways (Davidson & Chen 2004). A permissive effect of Proteobacteria on MTT levels can be hypothesised through the modulation of adrenal and hepatic cytochrome oxidases involved in drug metabolism (Kitamura *et al.* 2002). Intestinal microbiota metabolites have been demonstrated to differentially affect hepatic cytochrome P450s and drug transporters (Ueyama *et al.* 2005) involved anti-cancer drug clearance (Scripture *et al.* 2005). The intratumoural measurement of MTT levels and mechanistic *in vitro* studies are necessary to demonstrate the mechanisms by which the bacteria taxa, prevalent in ACC specimens, impact on MTT levels.

Although we found some significant correlations between the bacterial ACC and tumour parameters, and MTT levels, our findings do not establish any causal role between the oncobiome and the development and progression of cancer or whether the bacterial colonisation simply reflects the infection of the tumour.

For sure, the peculiar vasculature system of the adrenal gland, consisting of the presence of sinusoids and a portal system, together with the peculiar immunoprotective niche characterised by glucocorticoid production from the cortex, may favour the bacterial colonisation of this gland, especially in cancer conditions. Further studies comparing gut and adrenal microbiota profiles in ACC patients, as well as reporting the detection of bacteria in the bloodstream, would help in defining the mechanistic way of tissue colonisation. In previous analyses adopting an optimised 16S metagenomic sequencing pipeline, the bacterial 16S rRNA gene was reported in normal tissues in mice, including the brain, muscle, fat, heart and liver. In addition, Xiao *et al.* found bacteria in healthy mouse liver (Sun *et al.* 2023). Therefore, two important queries may come up: (i) where are the intratissue bacteria from and (ii) what are their functions? The process of gut leakage may be the most widely recognised answer to the first issue (Camilleri & Vella 2022). However, the study of Xiao raised another hypothesis for the origin of the microbiota in the healthy liver, according to which the liver microbiomes are inherent components of hepatocytes. However, it is also unknown what functions the liver microbiota serves. Healthy tissue bacteria may perform mostly metabolic tasks as opposed to those that endanger the host. We think that the origin of life may be relevant in terms of whether these endogenous bacteria are parasitic or symbiotic, as well as whether they are somehow involved in the control of gene expression or cell life activities (Camilleri & Vella 2022).

We recognise the major limitations of this study: (i) the limited number of samples, which nevertheless is relevant for a monocentric study on a rare cancer; (ii) the 16S sequencing has a limited ability to determine species identity and does not permit the study of microbial functional content; (iii) at this stage, our findings are mainly descriptive as we need larger cohorts of patients to consolidate the causal nature and study the bacterial functions investigating metabolic aspects of this cross-talk; (iv) despite the sterility of the overall workflow and the filters applied *in silico* to exclude contaminating DNA, some reads (e.g. Cyanobacteria) are possibly derived from bacterial DNA ubiquitous in extraction and amplification kits (Salter *et al.* 2014, Zhang *et al.* 2015).

## Conclusions

Our explorative study is the first to describe bacterial colonisation of the adrenal cortex, with a specific microbiota profile associated with ACC condition. Moreover, the differences in phyla and *genera* prevalence are associated with high levels of circulating MTT, in particular at 9 months from the treatment start, suggesting the existence of a peculiar oncobiome profile predisposing for MTT increase. Further studies on larger cohorts of ACC patients are required to (i) elucidate the active role of the infiltrating microbiota in regulating ACC development and progression and (ii) assess the potential development of novel selective immune anticancer approaches based on the specificity of the microbial bacterial products in the tumour.

## Declaration of interest

The authors declare that there is no conflict of interest that could be perceived as prejudicing the impartiality of the research reported.

## Funding

This work received support from EU Twinning European project: 952583—MICAfrica—H2020-WIDESPREAD-2018-2020/H2020-WIDESPREAD-2020-5 and University of Florence BANDO DI ATENEO PROGETTI ‘PROBLEM-DRIVEN’- PROGETTO FONZIE D.R.1116/2022 prot.202389). This publication was produced with the co-funding European Union - Next Generation EU, in the context of The National Recovery and Resilience Plan, Investment 1.5 Ecosystems of Innovation, Project Tuscany Health Ecosystem (THE), CUP: B83C22003920001.

## Data availability

The raw sequencing data are deposited in NCBI Gene Expression Omnibus under the accession number GSE228424. Further details about the microbiota data processing and analysis are available at https://github.com/LeandroD94/Papers/tree/main/2023_ACC_Surrenal_oncobiota.
